# The formation of pre-effectors in the steady state opens a new perspective for cancer immunosurveillance

**DOI:** 10.18632/oncotarget.27967

**Published:** 2021-06-22

**Authors:** Alexey Surnov, Daniel Hawiger

**Keywords:** Hopx, pre-effectors, steady state, cancer immunosurveillance, dendritic cells

Immunosurveillance refers to the identification and rejection of cancer cells by the immune system, and CD4^+^ T cells play an important role in this process [1, 2]. CD4^+^ T cells can become activated as early as 3 days after the experimental exposure to tumor antigens [3] and functions of such CD4^+^ T cells are crucial for priming of anti-tumor cytotoxic CD8^+^ T cells [4]. Importantly, immunosurveillance is expected to occur during the early stages of the tumor formation process in an environment that closely corresponds to the steady state, which is characterized by the absence of specific pro-inflammatory stimuli [1, 2]. Several mechanisms have been proposed to explain the initiation of T cell anti-tumor immune responses that lead to a subsequent elimination of malignant cells as reviewed in [5, 6]. For example, mechanisms that rely on “don’t eat me” signals, such as CD47, whose expression is decreased on cancer cells, facilitate a recognition of cancerous cells [7]. Further, developing tumors start producing specific markers that may indicate tissue damage [8, 9]. Overall, these and other similar mechanisms likely contribute to the early initiation of effector CD4^+^ T cell responses even in the absence of specific pro-inflammatory signals such as those dependent on tissue necrosis caused by tumors.

However, it has also been established that steady state conditions support the induction of immunological tolerance rather than priming of effector T cells (as recently reviewed in [10]). Under steady state conditions, antigen-activated CD4^+^ T cells are readily converted to peripherally induced regulatory T cells (pTreg cells) [10–12]. Therefore, the preferential induction of tolerance mechanisms in the steady state is not easily reconciled with a formation of specific effector T cells during immunosurveillance. However, our recently published results revealed that in addition to promoting a conversion into pTreg cells, antigen-specific activation of T cells in the steady state unlocks a much broader potential for subsequent T cell differentiation [13]. Specifically, we uncovered that in addition to the initiation of pTreg cell conversion, T cells can also become specifically “pre-committed” in the steady state to undergo Th1/Th17 effector differentiation. This two-step process involves a formation of specialized precursors that lack the expression of key transcriptional “master regulators” of T cell fate such as Foxp3, RORγt or T-bet. Instead, these precursors become imprinted with defined epigenetic and transcriptional programs that support subsequent terminal effector differentiation. Whereas a conversion into pTreg cells requires the continuous presence of tolerogenic signals, the imprinted programs promote effector differentiation even under minimally skewing conditions. This work has further revealed a specific expression in such precursors of Homeodomain only protein (Hopx) [13]. Hopx is a highly evolutionarily conserved small protein that is also present in some differentiated CD4+ effector, memory, and regulatory T cells [13–15]. However, Hopx has also been recently established as a novel marker of specific developmental potentials in various non-hematopoietic progenitor populations in human and mouse systems [15]. Our recent results revealed that the specific expression of Hopx marks T cells that have relevant epigenetic and transcriptional signatures but lack other established master regulators of T cell fate. In contrast, low expression of Hopx corelates with the absence of such pre-effector epigenetic and transcriptional signatures and instead indicates a capacity for a robust subsequent conversion into pTreg cells in the presence of additional tolerogenic signals as discussed above. Overall, the detection of expression of Hopx, combined with the identification of corresponding epigenetic and transcriptional signatures in such T cells, could become a powerful tool for the analysis of early developing anti-tumor effectors.

Conventional dendritic cells (cDC) are crucial for inducing the activation and subsequent differentiation of naïve T cells. Type 1 cDC (cDC1) are capable of presenting antigens to both CD4^+^ and CD8^+^ T cells. Therefore, cDC1 are crucial for anti-cancer immune responses [1, 16]. However, cDC1 also have powerful tolerogenic functions, whereas cDC2 are considered as more potent in inducing effector T cells [10]. We now found that in the steady state, antigen presentation by either cDC1 or cDC2 can induce in naïve T cells the specific expression of Hopx, which is indicative of the induction of the “pre-effector” epigenetic and transcriptional programs that pre-commit such T cells toward effector terminal differentiation Therefore, these new findings also help to clarify the dual roles of cDC1 that induce mechanisms of tolerance as well as promote initiation of immune responses even in the absence of specific skewing signals [13].

**Figure 1:**
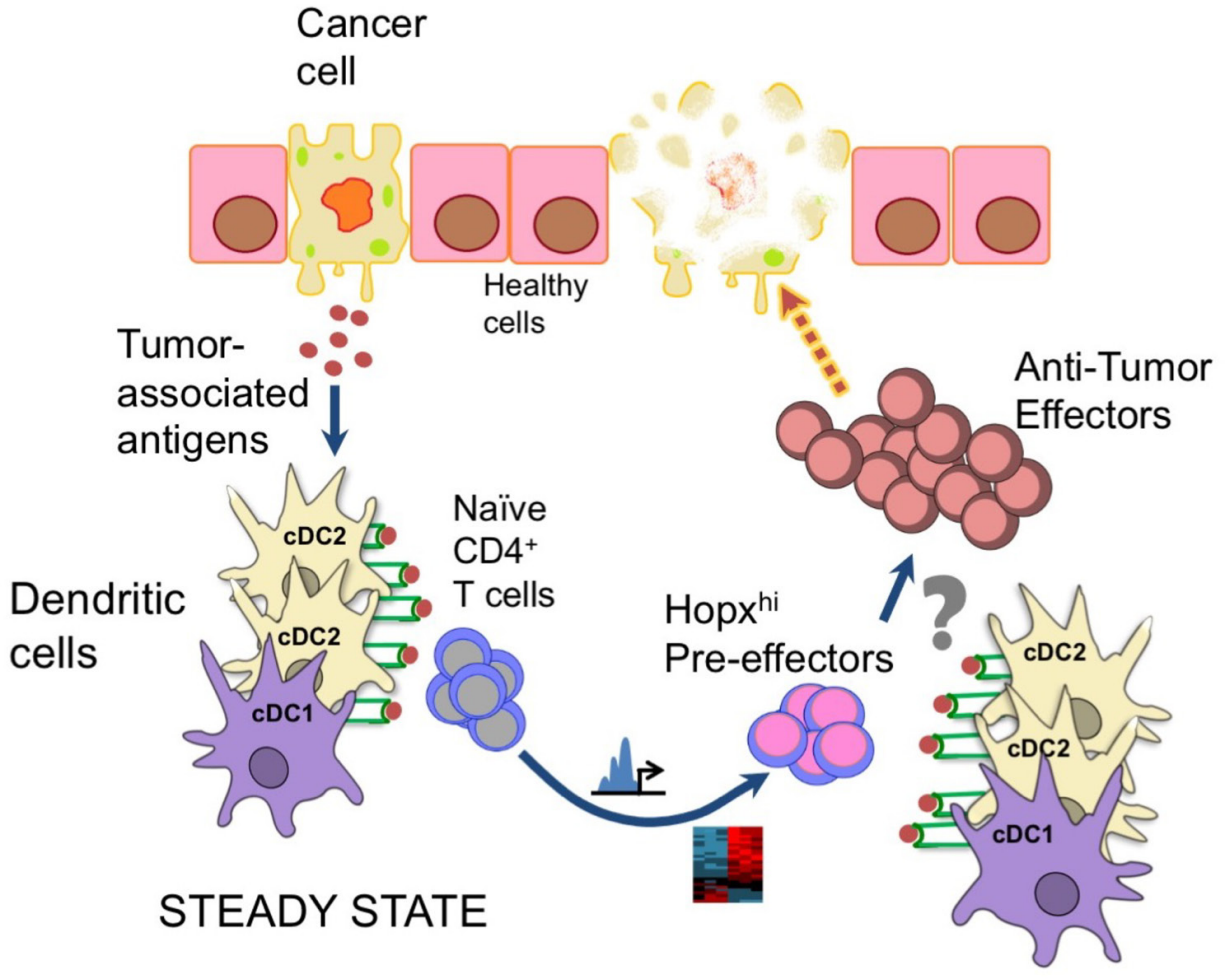
Hopx-expressing (Hopx^hi^) CD4^+^ pre-effectors are epigenetically and transcriptionally programmed in the steady state by dendritic cells in response to various antigens. These Hopx^hi^ pre-effectors can readily terminally differentiate into effectors. The mechanisms of differentiation of such effectors that could contribute to anti-tumor responses remain to be investigated.

Overall, we propose that the pre-effector CD4^+^ T cells may arise in response to tumor-associated antigens and contribute to the immune responses elicited against corresponding tumorous cells (Figure 1). Addtionally, the functions of specific cDC could be further exploited by delivering defined tumor antigens to cDC populations *in vivo* [17]. Therefore, the impact of such anti-tumor pre-effector CD4^+^ T cells on oncologic diseases will be of clear interest for further experimental and clinical studies.
